# Local Cytokine Release Syndrome After Idecabtagene Vicleucel Therapy in Patients With Multiple Myeloma: Two Case Reports

**DOI:** 10.7759/cureus.72364

**Published:** 2024-10-25

**Authors:** Taku Kikuchi, Nobuhiro Tsukada, Kodai Kunisada, Moe Nomura-Yogo, Tadao Ishida

**Affiliations:** 1 Department of Hematology, Japanese Red Cross Medical Center, Tokyo, JPN

**Keywords:** cervical edema, chimeric antigen receptor-t cell, dexamethasone, local cytokine release syndrome, multiple myeloma

## Abstract

Chimeric antigen receptor T-cell (CAR-T) therapy targeting the B-cell maturation antigen (BCMA) is an effective treatment for patients with relapsed/refractory multiple myeloma (RRMM). However, cytokine release syndrome (CRS) represents a significant complication associated with CAR-T therapy. While most CRS cases involve systemic symptoms such as fever, hypotension, and respiratory distress, localized symptoms (referred to as local CRS) can also occur. Herein, we report two cases of local CRS without cervical lesions that occurred at our institution.

Both patients had triple-class refractory RRMM prior to therapy. Following idecabtagene vicleucel (ide-cel) administration, both developed grade 1 CRS on the day of ide-cel administration; one case improved with tocilizumab, while the other improved with tocilizumab and dexamethasone (dex). However, on the third day post-administration, they exhibited symptoms characterized by neck swelling, leading to a risk of airway obstruction. Both cases were diagnosed as local CRS, and prompt dex administration resulted in rapid symptom improvement.

These cases underscore the importance of monitoring for local CRS even in the absence of "cervical myeloma lesions", particularly during the early phase following ide-cel administration. Early administration of dex is crucial for the effective management of local CRS.

## Introduction

Chimeric antigen receptor T-cell (CAR-T) therapy has shown promising results in patients with relapsed/refractory multiple myeloma (RRMM) [1-3]. However, it is associated with specific complications such as cytokine release syndrome (CRS) and immune effector cell-associated neurotoxicity syndrome (ICANS), which can be severe. While CRS typically presents with systemic symptoms such as fever, hypotension, and hypoxia, there have been rare reports of local CRS characterized by symptoms such as cervical edema [4-10]. In this report, we present two cases of local CRS that developed in patients with RRMM following the administration of idecabtagene and vicleucel (ide-cel) at our institution.

## Case presentation

Case 1

**Figure 1:**
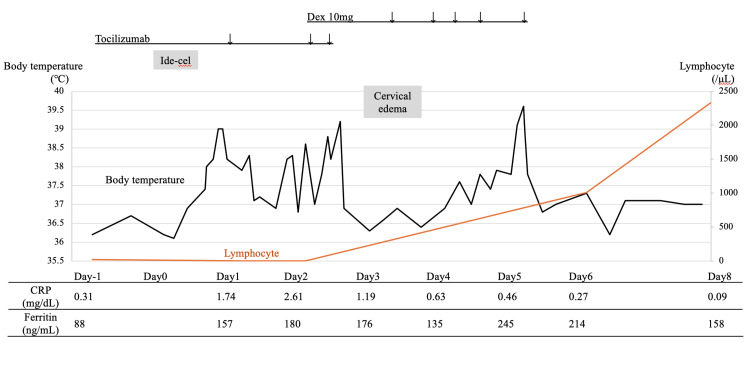
Clinical course of ide-cel administration. The black line indicates body temperature and the orange line represents lymphocyte count. Ide-cel was administered on day 0. Ide-cel: idecabtagene vicleucel; Dex: dexamethasone.

A 65-year-old woman was diagnosed with multiple myeloma (IgG-κ, International Staging System (ISS) I) 14 years before. Following the administration of induction therapy with bortezomib, thalidomide, and dexamethasone (dex), she received high-dose melphalan followed by autologous peripheral blood stem cell transplantation (ASCT), followed by two years of maintenance therapy with lenalidomide. Disease progression was observed one year after the maintenance therapy. Despite considerable treatment with five courses of ixazomib, lenalidomide, and dex, followed by three years of daratumumab, lenalidomide, and dex proved ineffective, and her disease evolved into triple-class refractory disease. Given triple-class exposure and history of three lines of prior therapy, she was eligible for treatment with ide-cel and underwent lymphocyte apheresis for its production. Bridging therapy with carfilzomib and dex (Kd) achieved a partial response (PR). Whole-body magnetic resonance imaging (MRI) before lymphodepletion chemotherapy (LDC) showed only bone lesions without any extramedullary disease, including in the "cervical myeloma lesions". Following LDC with fludarabine and cyclophosphamide, ide-cel (389.05 × 106 anti-BCMA CAR-T cells) was administered. The patient’s clinical course is shown in Figure 1.

**Figure 2:**
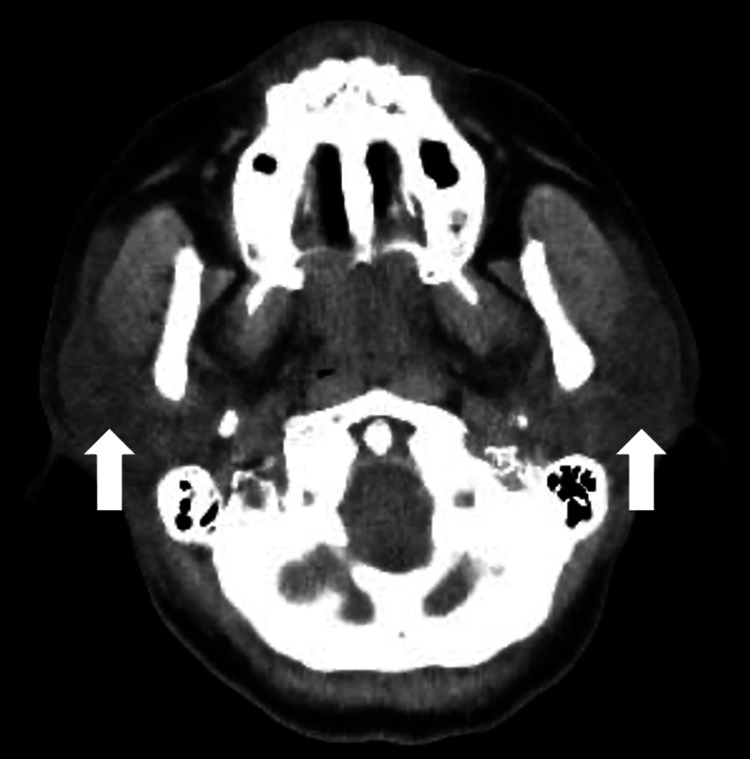
CT findings at the onset of local CRS, showing bilateral parotid gland swelling CT: computed tomography; CRS: cytokine release syndrome.

**Figure 3:**
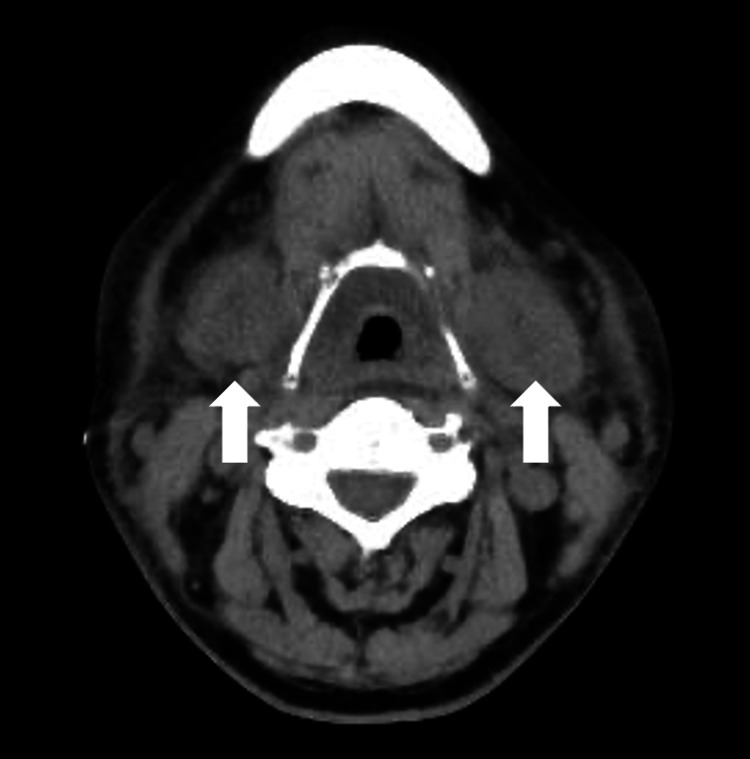
CT findings at the onset of local CRS, showing bilateral submandibular gland swelling. CT: computed tomography; CRS: cytokine release syndrome.

The patient developed fever on the day of ide-cel administration, and a diagnosis of grade 1 CRS was made. Tocilizumab (toci) was administered to treat the CRS. Although oxygen supplementation was required on day 2 post-ide-cel, the patient’s fever resolved, and her oxygen requirements decreased by day 3 after three doses of tocilizumab. However, on day 3, she developed cervical swelling and hoarseness, and CT findings revealed parotid and submandibular gland swelling, posing a risk of airway obstruction (Figures 2, 3).

Local CRS of the neck was diagnosed based on physical examination and CT findings. Dex (10 mg) was initiated, leading to rapid symptom improvement. Although transient fever recurred, there was no recurrence of local CRS after a total of 50 mg of dex was administered over three days. Bone marrow examination one month after CAR-T administration achieved a negative minimal residual disease.

Case 2

**Figure 4:**
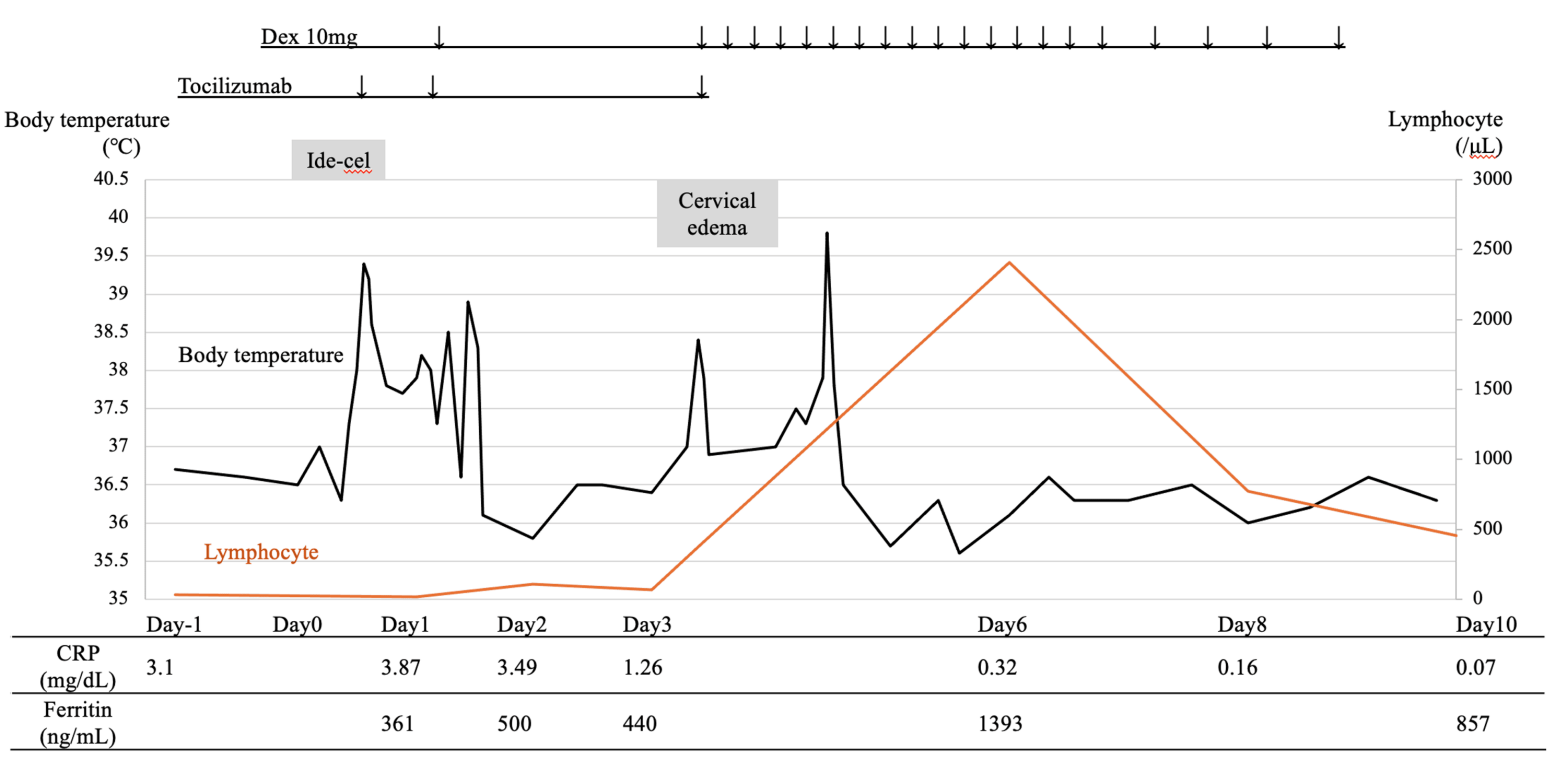
The clinical course of ide-cel administration. The black line indicates body temperature and the orange line represents lymphocyte count. Ide-cel was administered on day 0. Ide-cel: idecabtagene vicleucel; Dex: dexamethasone.

A 65-year-old man had been diagnosed with multiple myeloma (IgG-κ, R-ISS II) six years prior, following a vertebral compression fracture. He received induction therapy with bortezomib, cyclophosphamide, and dex, followed by ASCT. Despite maintenance therapy with lenalidomide, his disease progressed one-year post-ASCT. Subsequent treatments with isatuximab, pomalidomide, and dex, as well as a regimen of bortezomib, thalidomide, dex, cisplatin, doxorubicin, cyclophosphamide, and etoposide, were all ineffective. In the end, Kd proved effective, leading to a second ASCT, which progressed rapidly within three months post-transplant. Given triple-class exposure disease and five lines of prior therapy, he was eligible for ide-cel. Lymphocyte apheresis for ide-cel production was initially unsuccessful; however, a second attempt proved successful and the patient achieved PR with bridging therapy using Kd. Whole-body MRI before LDC showed only bone lesions without extramedullary disease, including in the "cervical myeloma lesions". Following LDC with fludarabine and cyclophosphamide, ide-cel (408.19 × 106 anti-BCMA CAR-T cells) was administered. The patient’s clinical course is shown in Figure 4.

**Figure 5:**
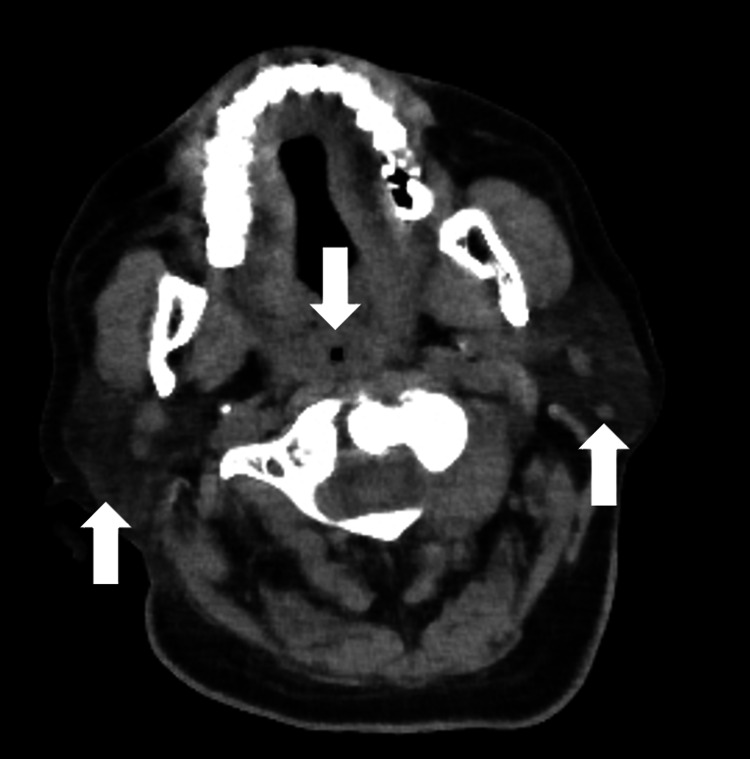
CT findings at the onset of local CRS, showing bilateral parotid gland swelling and airway stenosis. CT: computed tomography; CRS: cytokine release syndrome.

**Figure 6:**
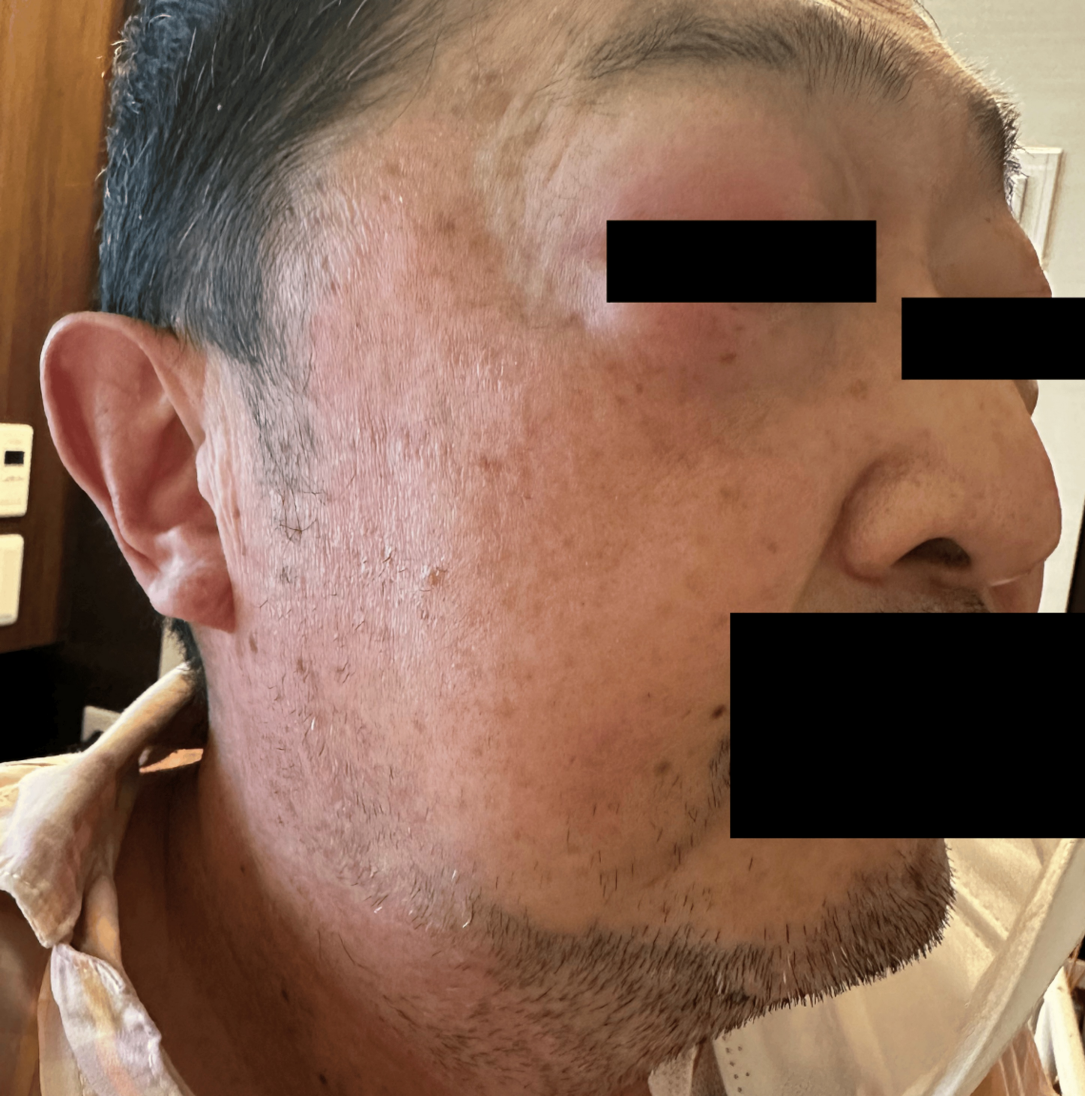
Clinical picture findings at the onset of local CRS, showing eyelid edema and neck swelling. CRS: cytokine release syndrome.

Fever was observed on the day of ide-cel administration, and tocilizumab was administered for grade 1 CRS, but the response was inadequate. Dex (10 mg) was therefore added. Although his fever resolved, the patient developed eyelid and cervical swelling on day 3 post-ide-cel administration. CT findings revealed bilateral parotid gland swelling and airway narrowing leading to a diagnosis of local CRS (Figures 5, 6).

**Figure 7:**
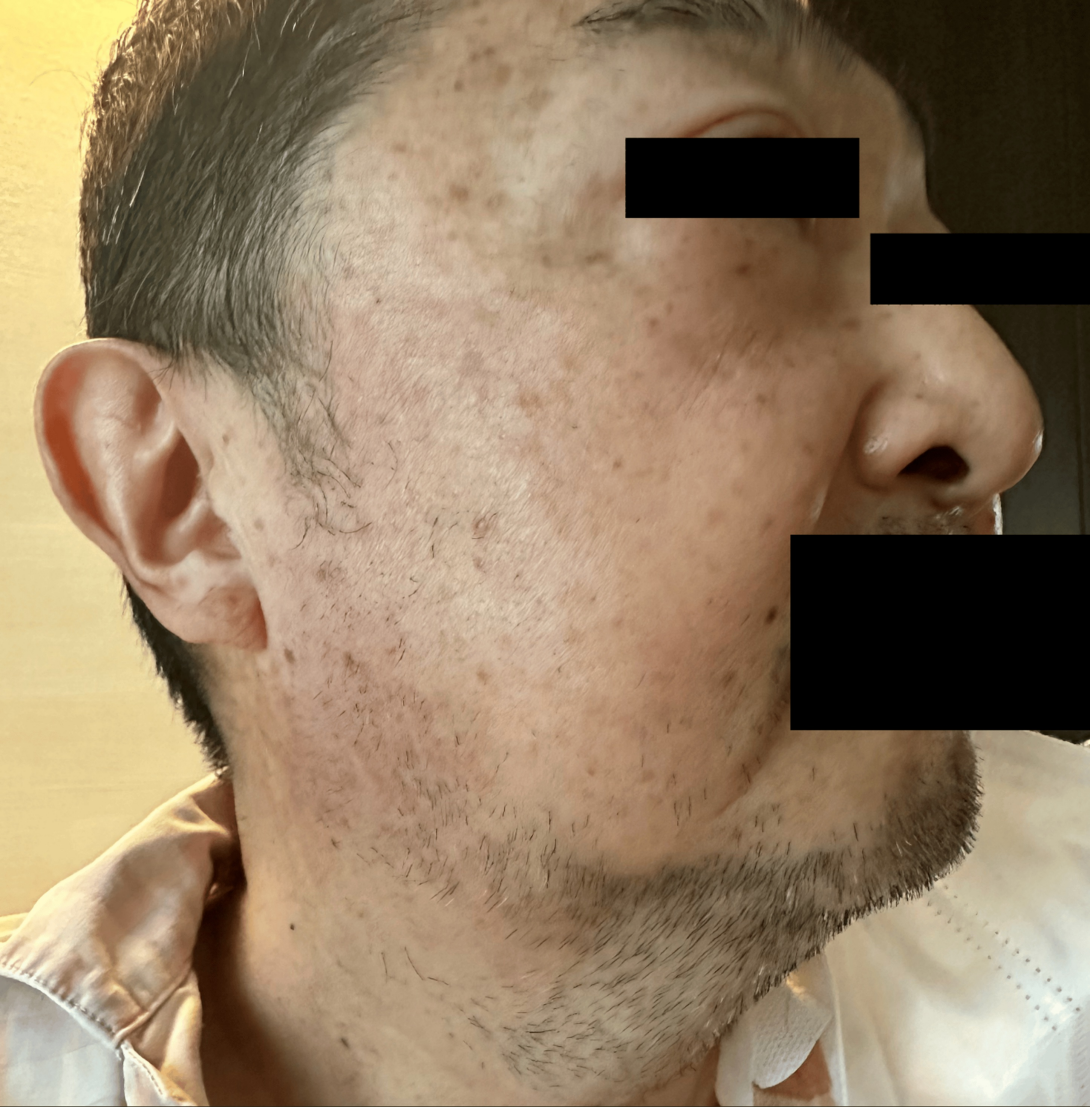
Clinical findings improved after dexamethasone administration

Oxygen saturation decreased and hoarseness was observed, and the patient reported breathing difficulty; therefore, dex (10 mg) was administered. His fever subsequently recurred, but his clinical symptoms improved following dex treatment, with no recurrence of local CRS (Figure 7). Bone marrow examination one month after CAR-T administration achieved a negative minimal residual disease.

## Discussion

Herein, we report two cases of local CRS following ide-cel administration in patients with RRMM, both of which responded rapidly to dex. In pivotal studies and real-world data, CRS of any grade has been reported to occur in 84% and 82% of patients, respectively, with grade ≥3 CRS occurring in 5% and 3% of patients, respectively [1,11]. However, the frequency of local CRS, characterized by cervical swelling, has not been well documented in the literature. Although the number of reports is limited, local CRS has been reported regardless of the underlying disease, as summarized in Table 1 [4-10].

**Table 1:** Characteristics of previous reported cases with local CRS after CAR-T therapy CAR; chimeric antigen receptor, CRS; cytokine release syndrome, CRP; C-reactive protein, ALL; acute lymphoblastic leukemia, DLBCL; diffuse large B-cell lymphoma, MM; multiple myeloma, Dex; dexamethasone, Toci; tocilizumab, mPSL; methylprednisolone, NA; not available

Authors	Reference	Age (years)	Sex	Primary disease	Type of CAR-T	CRS (grade)	Days to onset systemic CRS from CAR-T	Days to onset local CRS from CAR-T	Symptom	CRP (mg/dL)	Treatment	Outcome
													Nakanishi et al.	4	54	Female	MM	Idecabtagene vicleucel	1	2	4	Dyspnea, laryngeal edema	NA	Dex	Improved
Jin et al.	5	51	Male	DLBCL	NA	3	0 (12 hours)	4	Pharngeal obstruction, Cervical edema	NA	Toci, Dex	Improved
Ko et al.	6	20	Female	ALL	Tisagenlecleucel	1	0	4	Cervical edema	7.11	Dex	Improved
Shima et al.	7	15	Male	ALL	Tisagenlecleucel	1	1	3	Cervical edema	NA	mPSL, Toci, Dex	Improved
Inoue et al.	8	60	Male	DLBCL	Tisagenlecleucel	1	1	5	Cervical edema	1.21	Observation	Improved
Inoue et al.	8	70	Male	DLBCL	Tisagenlecleucel	1	2	3	Cervical edema	4.21	Dex	Improved
Luan et al.	9	61	Female	ALL	Tisagenlecleucel	2	0 (12 hours)	8	Facial edema, Cervical edema, Dyspnea	NA	mPSL, Dex	Improved
Kawase et al.	10	75	Male	DLBCL	Lisocabtagene maraleucel	1	2	5	Cervical edema Hypoxia	0.88	Dex	Improved
Kawase et al.	10	73	Male	DLBCL	Lisocabtagene maraleucel	1	1	4	Cervical edema Hypoxia	0.58	Dex	Improved
Kawase et al.	10	71	Female	DLBCL	Tisagenlecleucel	1	2	4	Cervical edema	0.22	Dex	Improved
Presented case 1	-	65	Female	MM	Idecabtagene vicleucel	1	0	3	Cervical edema Hypoxia	1.19	Dex	Improved
Presented case 2	-	65	Male	MM	Idecabtagene vicleucel	1	0	3	Facial edema, Cervical edema	0.97	Toci, Dex	Improved

Notably, all reported cases have come from Asia. All cases involved cervical swelling, and rapid improvement was observed with dex treatment, with all cases having systemic CRS preceding the local CRS. In cases where methylprednisolone was used for local CRS, it proved ineffective, and improvement was ultimately achieved only after dex administration [7]. Local CRS can lead to airway obstruction caused by cervical swelling. Although one case showed natural remission without dexamethasone administration if symptoms such as hoarseness and decreased oxygen saturation suggest a risk of airway obstruction, prompt administration of dexamethasone is recommended as an effective treatment for local CRS.

The previously reported case of local CRS following ide-cel administration involved a patient with thyroid plasmacytoma who developed local CRS after receiving ide-cel subsequent to radiation therapy for the thyroid [4]. By contrast, our two cases did not present with any "cervical myeloma lesions" associated with cervical edema. This finding suggests that the development of local CRS after ide-cel is not necessarily associated with the presence of "cervical myeloma lesions".

The duration from CAR-T administration to local CRS onset was three to five days in all but one case, indicating that neither the disease type nor CAR-T correlated with the timing of local CRS onset. In our cases, the onset of local CRS coincided with an increase in lymphocytes, suggesting a potential correlation between CAR-T expansion and local CRS onset. In other words, the expansion of CAR-T may be associated with the onset of local CRS, leading to temporary inflammation primarily in the cervical region. However, the exact mechanism of onset remains unclear. In situations requiring airway obstruction, it is often challenging; however, performing a pathological biopsy may lead to a more accurate understanding of the etiology. In addition, even after systemic symptoms improve and inflammation subsides, local CRS can still occur, highlighting the need for careful monitoring of clinical symptoms such as cervical swelling and mild hypoxia, particularly within the first week following CAR-T administration.

## Conclusions

Even in patients with RRMM without "cervical myeloma lesions", there is a risk of local CRS after ide-cel administration. Early signs such as neck swelling should be closely monitored, and early dex administration is crucial for effectively improving local CRS. Further investigations are warranted to elucidate the specific factors and interactions that lead to this syndrome in patients who are treated with CAR-T therapies.
